# Access to Artemisinin-Combination Therapy (ACT) and other Anti-Malarials: National Policy and Markets in Sierra Leone

**DOI:** 10.1371/journal.pone.0047733

**Published:** 2012-10-25

**Authors:** John H. Amuasi, Graciela Diap, Samuel Blay Nguah, Patrick Karikari, Isaac Boakye, Amara Jambai, Wani Kumba Lahai, Karly S. Louie, Jean-Rene Kiechel

**Affiliations:** 1 University of Minnesota School of Public Health, PhD Program, Division of Health Policy and Management, Minneapolis, Minnesota, United States of America; 2 Komfo Anokye Teaching Hospital (KATH), Kumasi, Ghana; 3 Drugs for Neglected Diseases initiative (DNDi), Geneva, Switzerland; 4 Ministry of Health and Sanitation, Freetown, Sierra Leone; 5 Department of Clinical Research, Faculty of Infectious & Tropical Diseases, London School of Hygiene and Tropical Medicine, London, United Kingdom; Kenya Medical Research Institute - Wellcome Trust Research Programme, Kenya

## Abstract

Malaria remains the leading burden of disease in post-conflict Sierra Leone. To overcome the challenge of anti-malarial drug resistance and improve effective treatment, Sierra Leone adopted artemisinin-combination therapy artesunate-amodiaquine (AS+AQ) as first-line treatment for uncomplicated *P. falciparum* malaria. Other national policy anti-malarials include artemether-lumefantrine (AL) as an alternative to AS+AQ, quinine and artemether for treatment of complicated malaria; and sulphadoxine-pyrimethamine (SP) for intermittent preventive treatment (IPTp). This study was conducted to evaluate access to national policy recommended anti-malarials. A cross-sectional survey of 127 medicine outlets (public, private and NGO) was conducted in urban and rural areas. The availability on the day of the survey, median prices, and affordability policy and available non-policy anti-malarials were calculated. Anti-malarials were stocked in 79% of all outlets surveyed. AS+AQ was widely available in public medicine outlets; AL was only available in the private and NGO sectors. Quinine was available in nearly two-thirds of public and NGO outlets and over one-third of private outlets. SP was widely available in all outlets. Non-policy anti-malarials were predominantly available in the private outlets. AS+AQ in the public sector was widely offered for free. Among the anti-malarials sold at a cost, the same median price of a course of AS+AQ (US$1.56), quinine tablets (US$0.63), were found in both the public and private sectors. Quinine injection had a median cost of US$0.31 in the public sector and US$0.47 in the private sector, while SP had a median cost of US$0.31 in the public sector compared to US$ 0.63 in the private sector. Non-policy anti-malarials were more affordable than first-line AS+AQ in all sectors. A course of AS+AQ was affordable at nearly two days’ worth of wages in both the public and private sectors.

## Introduction

The West African country of Sierra Leone ranks as one of the least developed and poorest countries in the world, with three-fourths of the 6 million population living below the poverty line of <US$1 per day [1]. In 2002, the country emerged from an 11-year civil war which collapsed much of the existing economy and health care infrastructure. Malaria remains a major communicable disease in post-conflict Sierra Leone with an estimated 1.3 million new cases and 1,734 reported number of deaths in 2009 [2]. In addition, among children under-five, malaria is the number one cause of death, responsible for two out of five child deaths. Transmission of malaria occurs all year round with endemicity ranging from mesoendemic to hyper/holoendemic and the prevalence rate of infection is estimated to be about 65%. Parasite distribution is mainly *Plasmodium falciparum* (90%) with mixed infections occurring occasionally with *Plasmodium malariae* and *Plasmodium ovale* [3].

Effective treatment with artemisinin-based combination therapy (ACT) has been central to malaria control efforts in Sierra Leone. With evidence of high resistance to chloroquine of up to 79% of malaria cases in 2004 [4], Sierra Leone changed their malaria control policy from chloroquine to artesunate-amodiaquine (AS+AQ) as first-line treatment for uncomplicated malaria, with artemether-lumefantrine (AL) as the alternative in cases of contraindications or adverse side effects. Quinine is recommended for treatment of severe malaria, with artemether (AM) as an alternative when quinine is not effective or contraindicated. Sulphadoxine pyrimethamine (SP) is used for intermittent preventive treatment in pregnancy (IPTp) [5]. In addition, the government of Sierra Leone has recently introduced a new fixed dose combination (FDC) of ASAQ tablets, taken over 3 days, to treat uncomplicated malaria, with the aim of improving prescribing practices, patient compliance, and to reduce the risk of parasitic resistance to AS+AQ. For the most vulnerable population of children under age five, only one tablet of the FDC ASAQ needs to be taken per day.

Since the adoption of AS+AQ, Sierra Leone has observed a marked decrease in mortality from 1.7 deaths per 10,000 population per day in 2005 to 1 death per 10,000 population per day in 2007 [6]. However, there is limited evidence of a sustained decrease in the number of malaria cases [5]. Despite supposed widespread ACT implementation, it is estimated that only 30% of children <5 years with fever and 9.1% of overall reported cases of malaria received any anti-malarial treatment, the proportion of which were ACTs is unknown [7].

In April 2009, over 900,000 expired doses of AS+AQ valued at of over US$ 500,000 were destroyed in Sierra Leone [8], [9]. The destruction of these drugs when there were severe shortages at the local level suggests poor drug forecasting and weakness in the distribution system, highlighting systemic problems within the health system. At present, policy-recommended anti-malarial treatments are supposed to be provided free of charge in all public and NGO facilities in Sierra Leone, and although ACT implementation has been scaled up nationwide it is unclear whether these treatments are available, accessible and affordable to those who need them.

The objective of this study was to evaluate the accessibility of national policy recommended anti-malarials, ACTs, quinine and SP according to the pricing, availability, and affordability in the public, private and non-governmental organization (NGO) health sector markets in Sierra Leone. Results from this study will help inform advocacy campaigns and policy recommendations to improve country-wide accessibility of the new-fixed dose ASAQ and other policy antimalarials to treat uncomplicated malaria.

## Methods

### Study Area

**Figure 1:**
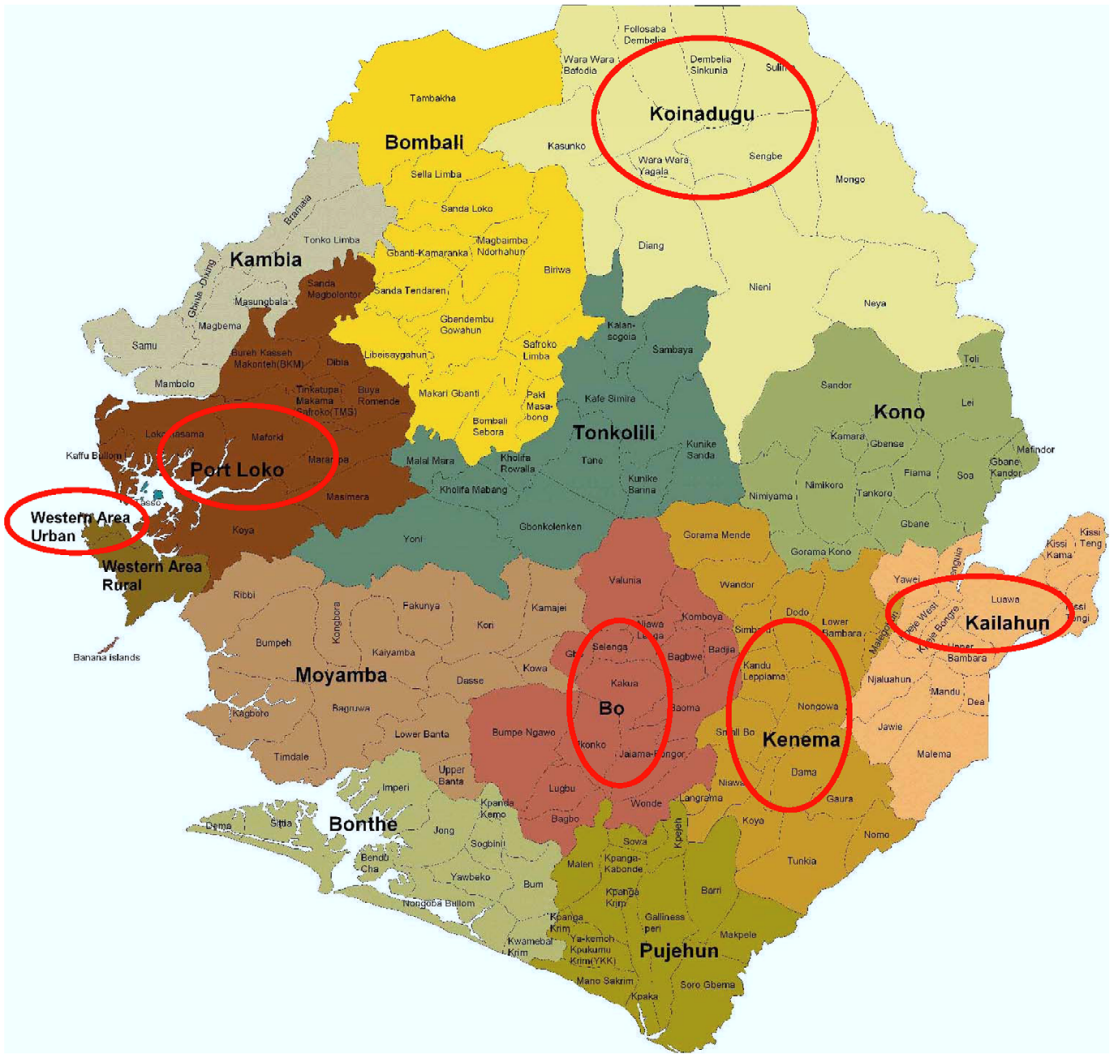
Anti-malarial market survey districts in Sierra Leone.

Adapting a standardized methodology developed by Health Action International/World Health Organization (HAI/WHO), a cross-sectional survey was conducted from 24 May to 3 June 2009 in six of the fourteen districts in Sierra Leone. The fourteen districts were first screened based on being reachable within one day’s travel from the country’s capital city, Freetown. This led to the exclusion of only one district – Bonthe. The six finally selected were: **Western area** - includes the capital city, Freetown; **Bo**: the second largest financial district in Sierra Leone, a districtwhere Médecins Sans Frontières (MSF) has had a long-term presence and runs a clinic. **Koinadugu**: has the largest chiefdoms, but is the most sparsely populated district, with 33% living within a 5km radius of a health facility, as opposed to the Kailahun, the second most sparse, with 64% of the population within reach of healthcare; **Kailahun**: the furthest district away from the capital city (12 hours travel time); **Port Loko** and **Kenema**: randomly selected electronically from the remaining nine districts. The selected areas also reflect urban (Western Urban Area and Bo) and rural (Kalihun, Kenema, Koinadugu, and Port Loko) populations (Figure 1).

### Selection of Medicine Outlets

Medicine outlets were selected from the public, private, and NGO sectors. Public medicine outlets were selected from a list compiled from the Ministry of Health and Sanitation (MoHS) with support from a MoHS project advisor. The public medicine outlets were selected using a two-stage sampling approach. First, from each of the six survey districts, the main public hospital was selected. Secondly, the remaining six public health facilities had their own pharmacies or dispensaries and were chosen if they were located within three hours’ travel from the main hospital. A matching number of private and NGO sector medicine outlets were selected which were geographically close to each public medicine outlet. In Kailaihun (n = 5), Kenema (n = 4), Koinadugu (n = 2) and Port Loko (n = 1) where there were fewer NGO outlets, we oversampled public and private medicine outlets to ensure an almost equal number of medicine outlets were surveyed in each district. All private medicine outlets in Port Loko (n = 5) and the Western Urban Area (n = 6) districts were sampled.

### Survey Methodology

Adapted WHO and Health Action International (HAI) standardised methodologies for surveying medicine prices, availability, and affordability were used for this survey [10]. Pharmacists or medicine dispensers were recruited to participate in the survey and verbal consent was obtained from those who agreed to participate. For each anti-malarial found in the medicine outlet on the day of the survey, trained data collectors recorded information on the brand name (originator and generic), price, manufacturer, and manufacturing company were recorded, as well as information on the medicine package type (loose pills, single pack, or hospital package), the availability of the anti-malarial as over-the-counter or prescription, and the number of anti-malarials sold in the last month. In addition, data on the medicine retailer’s knowledge regarding WHO recommendations and national anti-malarial treatment guidelines were collected. The prices were originally recorded in Sierra Leonean Leones (SLL) and then converted to US dollars (US$). At the time of the survey, US$1 was equivalent to SLL3,200.

### Data Analysis

Data from the different sector medicine outlets were analysed separately, and anti-malarials were stratified by treatment policy: first-line ACT (AS+AQ or artemether-lumefantrine (AL) according to Sierra Leonean’s national policy); second-line artemether or quinine; intermittent preventive treatment in pregnancy (IPTp) with SP; and other non-policy anti-malarials. For the availability analysis, different dosage strengths of the same anti-malarial were combined to calculate the overall availability of that anti-malarial. Median prices of anti-malarials were computed if there were ≥4 medicines (following the WHO-HAI methodology) identified for each anti-malarial category regardless of dosage or form; otherwise the mean was calculated [10]. Anti-malarials offered for free were excluded from the price analysis.

Affordability was estimated using median anti-malarial prices and the average per capita annual income (US$320/per year or US$0.88/day) in Sierra Leone [11] and calculating the number of days’ wages required to purchase a course of the anti-malarial.

## Results

In this study, a total of 127 medicine outlets, of which 57 (44.9%) public sector hospitals, 44 (34.6%) private medicine outlets, and 26 (20.5%) NGO medicine outlets were surveyed in the regions of Bo (n = 22), Kailahun (n = 22), Kenema (n = 21), Koinadugu (n = 26), Port Loko (n = 16), and Western Urban Area (n = 20), respectively.

### Availability of Anti-malarials

At the time of survey, all public medicine outlets, 97.7% of private and 88.5% of NGO medicine outlets stocked anti-malarials. (Table 1). Public medicine outlets (96.5%) were more likely to stock AS+AQ and AL for than NGO (76.9%) and private (56.8%) medicine outlets. AL was only found in private and NGO outlets. Quinine was found in nearly two-thirds of both public and NGO outlets but in only in about one-third of private outlets. The oral quinine tablet was more common than its injection formulation across all sectors. Artemether, an alternative to quinine, was three times more available in the private and NGO outlets than the public outlets. SP for IPTp was available in three-quarters of the public outlets and over half of both private and NGO outlets. Over 80% of private sector outlets stocked non-policy anti-malarials compared to 30% and 5% of NGO and public outlets, respectively. Nine types of non-policy anti-malarials were found in the market, of which two were ACTs (artesunate-mefloquine and artesunate-sulfadoxine pyrimethamine). Although much of Sierra Leone is known to be chloroquine-resistant, the drug was stocked in 70% of private outlets compared to none in public outlets. Almost half of both public and NGO outlets carried all three types of policy anti-malarials for treatment of uncomplicated malaria, complicated malaria and IPTp, whereas only one-quarter of private outlets stocked them.

**Table 1:** Availability of antimalarials in the public, private, and charitable health facilities surveyed.

	Health sector					
	Public (n = 57)	%	Private (n = 44)	%	NGO (n = 26)	%
**First-line ACT policy antimalarials for uncomplicated malaria**	**55**	**96.5**	**25**	**56.8**	**20**	**76.9**
Artesunate-amodiaquine	55	96.5	25	56.8	20	76.9
Artemether-lumefantrine	–	–	2	4.5	1	3.8
**Policy anti-malarials for complicated malaria**	**37**	**64.9**	**17**	**38.6**	**17**	**65.4**
Quinine injection	15	26.3	9	20.5	8	30.8
Quinine tablets	31	54.4	10	22.7	14	53.8
Artemether (Injection, suspension, tablets)	3	5.3	6	13.6	4	15.4
**Sulphadoxine-pyrimethamine for IPTp**	**44**	**77.2**	**24**	**54.5**	**16**	**61.5**
**Non-policy antimalarials**	**3**	**5.3**	**35**	**79.5**	**8**	**30.8**
Artesunate-mefloquine^a^	–	–	1	2.3	–	–
Artesunate-sulfadoxine pyrimethamine^a^	–	–	5	11.4	1	3.8
Amodiaquine (Suspension, Tablets, Injections)	1	1.8	6	13.6	1	3.8
Artesunate (Suppository and tablets)	2	3.5	10	22.7	3	11.5
Chloroquine (Injection, syrup, tablets)^b^	–	–	31	70.5	4	15.4
Halofantrine (Syrup and tablets)	–	–	3	6.8	–	–
Mefloquine^b^	–	–	2	4.5	1	3.8
Proguanil	–	–	1	2.3	–	–
Pyrimethamine	–	–	1	2.3	–	–
**Summary of available antimalarials**						
First-line policy ACT for uncomplicated malaria only	3	5.3	4	9.1	3	11.5
Policy anti-malarials for uncomplicated and complicated malaria	37	64.9	12	27.3	14	53.8
Policy anti-malarials for uncomplicated and complicated malaria and IPTp	27	47.4	11	25.0	12	46.2
Non-policy antimalarials only	0	0.0	8	18.2	0	0.0
No antimalarials	0	0.0	1	2.3	3	11.5

ACT, artemisinin-combination therapy; IPTp, intermittent preventive treatment in pregnancy.

a Other artemisinin-based combination therapy (ACT)

b Former first-line treatment policy antimalarial.

In general, urban areas (Bo and Western Urban Area) were more likely to stock policy anti-malarials than rural areas (Kailahun, Kenema, Koinadugu, and Port Loko) in all outlet types, in addition to being more likely to carry all three types of policy antimalarials (Table 2). Irrespective of being in an urban or rural area, the private sector was less likely to carry policy anti-malarials than the public and NGO sectors. Stocking non-policy anti-malarials was two-fold higher in the rural areas than the urban areas. One-quarter of private rural medicine outlets carried non-policy anti-malarials solely. Reassuringly, all medicine outlets in rural areas carried some anti-malarials, whereas, no anti-malarials were found in one private and 3 NGO outlets in the urban areas surveyed.

**Table 2:** Availability of antimalarials by urban vs. rural areas and Sector Type.

Urban areas (Bo and Western Urban Area)	Public (n = 14)	%	Private (n = 14)	%	NGO (n = 14)	%
**Policy antimalarials**						
First-line ACTs for uncomplicated malaria (artesunate-amodiaquine andartemether-lumefantrine)	14	100.0	8	57.1	10	71.4
Policy antimalarials for complicated malaria (quinine and artemether)	12	85.7	8	57.1	10	71.4
Sulphadoxine-pyrimethamine for IPTp	10	71.4	10	71.4	8	57.1
**Non-policy anti-malarials**	2	14.3	10	71.4	4	28.6
**Summary**						
First-line policy ACT for uncomplicated malaria only	0	0.0	1	7.1	1	7.1
Policy anti-malarials for uncomplicated and complicated malaria	12	85.7	6	42.9	9	64.3
Policy anti-malarials for uncomplicated and complicated malaria and IPTp	8	57.1	6	42.9	8	57.1
Non-policy antimalarials only	–	–	–	–	–	–
No antimalarials	–	–	1	7.1	3	21.4
**Rural areas (Kalihun, Kenema, Koinadugu, and Port Loko)**	**Public (n = 43)**	**%**	**Private (n = 30)**	**%**	**NGO (n = 12)**	**%**
**Policy antimalarials**						
First-line ACTs for uncomplicated malaria(artesunate-amodiaquineand artemether-lumefantrine)	41	95.3	17	56.7	10	83.3
Policy antimalarials for complicated malaria (quinine and artemether)	25	58.1	9	30.0	7	58.3
Sulphadoxine-pyrimethamine for IPTp	34	79.1	14	46.7	8	66.7
**Non-policy antimalarials**	1	2.3	25	83.3	4	33.3
**Summary**						
First-line policy ACT for uncomplicated malaria only	3	7.0	3	10.0	2	16.7
Policy anti-malarials for uncomplicated and complicated malaria	25	58.1	6	20.0	5	41.7
Policy anti-malarials for uncomplicated and complicated malaria and IPT	19	44.2	5	16.7	4	33.3
Non-policy antimalarials only	0	0.0	8	26.7	0	0.0
No antimalarials	0	0.0	0	0.0	0	0.0

ACT, artemisinin-combination therapy; IPTp, intermittent preventive treatment in pregnancy.

### Medicine Prices

**Figure 2:**
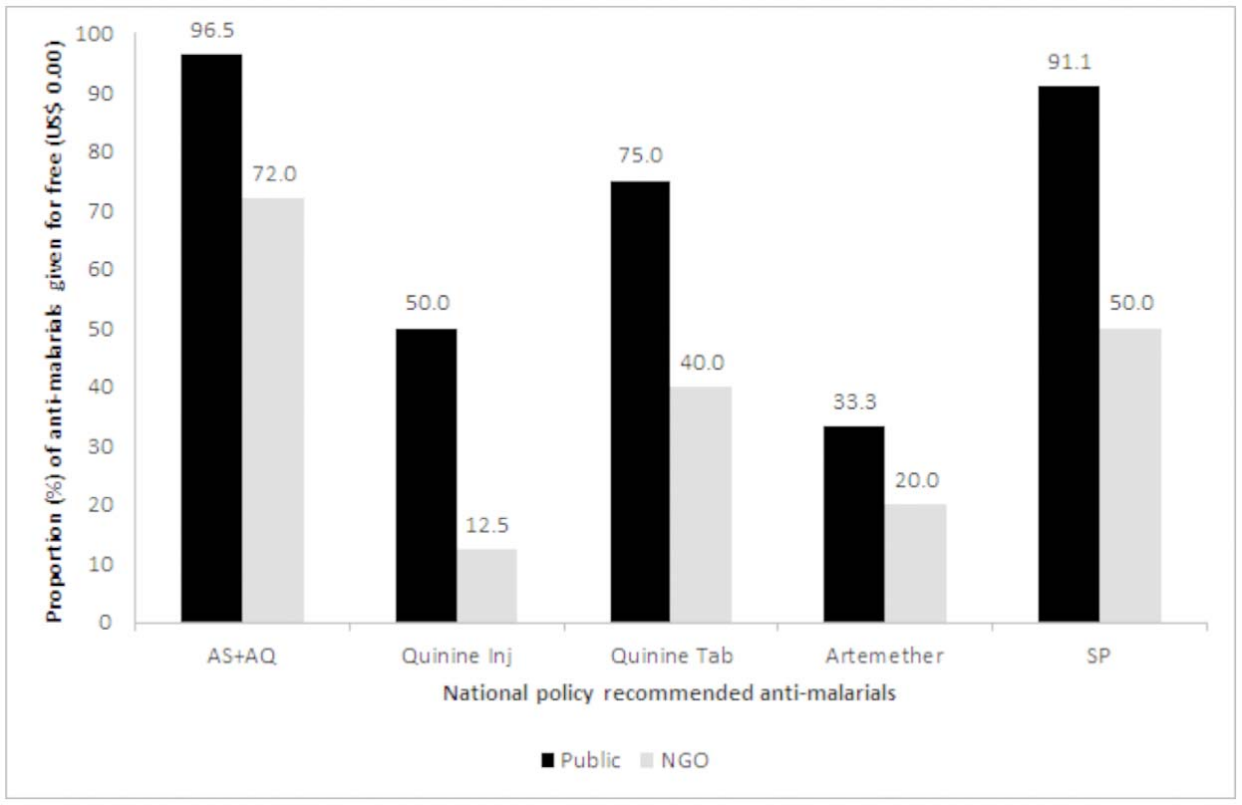
Proportion of national policy recommended anti-malarials offered for free ($USD 0.00) in the public and NGO sector medicine outlets surveyed in Sierra Leone. Artemether-lumefantrine was not found in the public sector and was not offered for free in the NGO sector.

Figure 2 shows the proportion of anti-malarials that were dispensed for free in the public and NGO sectors in line with national policy. All policy recommended anti-malarials were more likely to be offered for free in the public sector compared to the NGO sector. Table 3 shows the number and median price of anti-malarials identified in the market. The median price for AS+AQ was US$1.56 in both the public and private sectors, which was three-times the cost of that found in the NGO sector (US$0.31). Artemether-lumefantrine was not available in the public sector, and varied widely from US$0.94 in the NGO sector to US$8.59 in the private sector. The median price of quinine injection was 1.5 times higher in the private sector compared to the public and NGO sectors, whereas the median price of quinine tablets (US$0.63) was the same in all three sectors. Artemether cost three- to four-fold higher in the public and private sectors, respectively, than the NGO sector. SP was priced the same in the public and NGO sectors (US$0.31) and two times higher in the private sector (US$0.63). The prices of non-policy antimalarials ranged between US$0.28–0.94 in the public sector, US$0.31–5.31 in the NGO sector, and US$0.09–12.50 in the private sector.

**Table 3:** Median prices of antimalarials being sold in the public, private and charitable sectors.

					Health sector					
		Total no. free antimalarials ($0)*		Overall	Public		Private		NGO	
	No. of antimalarials	n	%	$USD§	$USD		$USD		$USD	
**First-line ACT policy antimalarials for uncomplicated malaria**	**144**	**100**	**69.4**	**1.56**	**1.56**	**(1.56–2.50)**	**1.56**	**(0.16–9.38)**	**0.47**	**(0.16–5.31)**
Artesunate-amodiaquine	141	100	70.9	1.56	1.56	(1.56–2.50)	1.56	(0.16–7.81)	0.31	(0.16–5.31)
Artemether-lumefantrine	3	–	–	6.04^a^	–		8.59	(7.81–9.38)^a^	0.94	
**Policy antimalarials for complicated malaria**	**113**	**41**	36.3	**0.47**	**0.31**	**(0.16–3.75)**	**0.63**	**(0.76–7.81)**	**0.31**	**(0.09–1.56)**
Quinine injection	33	9	27.3	0.31	0.31	(0.16–0.94)	0.47	(0.16–1.56)	0.31	(0.09–0.31)
Quinine tablets	58	30	51.7	0.63	0.63	(0.31–0.94)	0.63	(0.63–1.88)	0.63	(0.31–1.25)
Artemether	22	2	9.1	2.66	3.13	(2.50–3.75)	4.69	(0.94–7.81)	1.25	(0.31–1.56)
**Sulphadoxine-pyrimethamine IPTp**	**91**	**49**	**53.8**	**0.47**	**0.31**	**(0.28–0.31)**	**0.63**	**(0.31–2.34)**	**0.31**	**(0.16–0.47)**
**Non-policy antimalarials**	**191**	**53**	**27.7**	**0.94**	**0.39**	**(0.28–0.94)**	**0.78**	**(0.09–12.50)**	**0.47**	**(0.31–5.31)**
Artesunate-mefloquine	1	–	–	10.94	–		10.94^b^		–	
Artesunate-sulfadoxine pyrimethamine	8	–	–	6.25	–		7.03	(3.75–8.75)	5.31^b^	
Amodiaquine	9	1	11.1	0.70	0.47^b^		0.94	(0.31–1.56)	–	
Artesunate	23	1	4.3	3.44	0.94^b^		3.59	(1.56–7.81)	1.56	(0.31–5.31)
Chloroquine	58	1	1.7	0.63	–		0.47	(0.09–2.19)	0.63	(0.63–0.94)
Halofantrine	5	–	–	10.00	–		10.00	(9.38–12.50)	–	
Mefloquine	3	1	33.3	1.56	–		1.56^b^		–	
Proguanil	1	–	–	0.94	–		0.94^b^		–	
Pyrimethamine	1	–	–	0.31	–		0.31^b^		–	

ACT, artemisinin-combination therapy; $USD, United States Dollar.

* Antimalarials that were offered for free were excluded from these median price estimates.

§ 1 United States Dollar was equivalent to 3200 Sierra Leone Leones.

a The mean was calculated following WHO-HAI methods which indicate there should be at least ≥4 medicines to calculate the median.

b The price of the one antimalarial that was found in the charitable sector is reported.

c IPTp (Intermittent Preventative Treatment in pregnancy).

### Affordability

Quinine for treatment of complicated malaria and SP for IPTp were more affordable than first-line AS+AQ in all sectors when they were being sold in the market, costing about 1 day or less worth of wages (Table 4). Where AS+AQ was not offered for free in the public outlets, it was just as expensive as in the private outlets, totalling nearly two days’ worth of wages. AL was the least affordable anti-malarial found, costing nearly ten days’ worth of wages in the private sector.

**Table 4:** Affordability of policy and non-policy antimalarials when purchasing the medicines in the public, private, and charitable sectors.

	Number of days wages		
Anti-malarial	Public sector	Private sector	NGO sector
Policy recommended anti-malarials			
Artesunate-amodiaquine	1.8	1.8	0.5
Artemether-lumefantrine	–	9.8	1.1
Quinine injection	0.4	0.5	0.4
Quinine tablets	0.7	0.7	0.7
Artemether	3.6	5.3	1.4
Sulphadoxine-pyrimethamine	0.4	0.7	0.4
Non-policy antimalarials	0.4	0.9	0.5

* A days’ wage in Sierra Leone is USD 0.88 (2,816 SLL).

## Discussion

This is the first study to evaluate the accessibility of national policy-recommended artemisinin combination therapy (ACT), quinine, SP, and other anti-malarials in the public, private, and NGO sectors of Sierra Leone. The results of this study show high availability of policy recommended anti-malarials in public medicine outlets in Sierra Leone with over 95% of public medicine outlets stocking first-line AS+AQ for treatment of uncomplicated malaria. Although quinine and artemether for treatment of complicated malaria and SP for IPTp were less available than AS+AQ in the public sector, they were more likely to stock these policy anti-malarials than the NGO and private sectors. The relatively high availability of AS+AQ in Sierra Leone compared to other post-conflict African countries who have also adopted AS+AQ as first-line treatment(76.3% in the Democratic Republic of Congo [12] and 87.5% in Burundi [13]) may be attributed to the government’s free pricing policy. Although policy anti-malarials, AS+AQ, quinine and SP, are supposed to be offered for free at all public and NGO medicine outlets through the Essential Drugs programme, only about half of the outlets carried them on the day they were surveyed. The availability of policy anti-malarials was lower than what was found in Burundi, where nearly 80% of all sectors carried policy anti-malarials, AS+AQ and quinine [13].

In this study, chloroquine was not found in any of the public medicine outlets, highlighting its successful ban in this sector. However alarmingly, banned chloroquine was the most common anti-malarial stocked in 70% of all private sector outlets. This differs significantly from Burundi, where chloroquine has been successfully banned in all public, private and NGO markets [13]. The dominant chloroquine stock in Sierra Leone reflects the number of challenges within the drug regulatory system which may result from the lack of effective monitoring and surveillance programs to enforce the ban and oversee quality assurance. Firstly, there are thirty-seven anti-malarials registered with the Pharmacy Board that are authorized to be marketed and sold in Sierra Leone (personal communication, Registrar, Pharmacy Board of Sierra Leone, 2009) but over 60 brands were identified in the market, which does not reflect the national policy on recommended anti-malarials. The persistent availability of unregistered medicines is worrying and potentially detrimental to efforts at ensuring the uptake of policy recommended anti-malarials by the Sierra Leonean population. Secondly, a lack of human resources and capacity to enforce drug regulations, leads to smuggling and peddling of substandard and counterfeit medicines throughout sub-Saharan Africa [14] For example, there are more than 1,000 border crossing points in Sierra Leone that are not secured by custom officials, making monitoring of drug imports almost impossible. In order to minimize the smuggling and peddling of substandard and counterfeit medicines and to monitor drug safety and efficacy, the WHO has supported the establishment of a Pharmacovigilance Unit in order to strengthen the operations of the Pharmacy Board [15].

Thirdly, increasing access to AS+AQ and other policy recommended anti-malarials may be difficult to achieve since 83% of the hard-to-reach rural retailers stocked non-policy anti-malarials which were offered at a much cheaper price than ACTs. Specifically, Koinadugu accounted for one-third of the chloroquine stocks identified in the survey. This is consistent with other studies where the price of ACTs is nearly 10 to 40 times higher than chloroquine [16]. Private medicine retailers may view selling chloroquine as more profitable since it is an affordable option for patients and provides a potentially larger market for the retailer. In public medicine outlets where AS+AQ was dispensed at a charge, the price was the same as that found in the private sector. These high costs may reflect Sierra Leone’s complete dependency on imported medicines since there is no local pharmaceutical manufacturing company operating in the country; and there is a complex drug distribution with mark-ups aimed at supporting the existing cost recovery regime [17].

Private medicine sellers may not have received adequate training on malaria treatment and management, as 84.2% of public sector medicine sellers in this study were familiar with the indication for ACT use as being for uncomplicated malaria, compared to 48.3% in the NGO and 20.5% in the private sectors. The availability of ineffective anti-malarials and irrational drug use make it difficult to ensure patient adherence to anti-malarial treatment protocols. Adequate education of medicine sellers and health workers managing patients with malaria is needed to enable successful and effective implementation of national anti-malarial policy in Sierra Leone. Furthermore, increased information, education and communication (IEC) programs are needed for the patient population to inform and empower them with knowledge, which will discourage dispensing of inappropriate drugs for treatment.

ACTs were not available in one-quarter of NGO medicine outlets and two public outlets indicating that there were possible stock-outs on the day of the survey. The frequency of stock-outs occurring is unknown. However, these stock-outs may be linked to the country’s irregular performance in obtaining Global Fund grants for malaria control and the poor logistical coordination among those involved in the provision of ACTs [6]. Further investigation is needed to determine if stock-outs are truly occurring and if so, at what point of the distribution system the problems may emanate from. These reasons, coupled with the weak capacity of the government to monitor the drug distribution system in Sierra Leone may have resulted in medicine outlets purchasing medicines from other sources.

The inadequate stocks and the high fees patients pay for medical consultation in the public system [6] may be forcing patients to buy their anti-malarials from the private sector where they are insufficiently stocked. Although the public sector stocks appropriate anti-malarials, the most vulnerable population do not access them since only 12% of Sierra Leonean children suspected of having malaria use public health services within 24 hours of the onset of a fever [6]. If prompt treatment is to be achieved, the lack of use of health services needs to be addressed Moreover, a number of stakeholders have suggested the need to formulate a specific policy leading to a centralized anti-malarial procurement system whereby government, NGOs, and donors can procure/donate anti-malarials based on the demand declared by the Ministry of Health. The feasibility of this policy needs to be further explored as it will allow both public and private sectors to access appropriate recommended stocks of anti-malarials from a central deposit.

Although public (primary and secondary healthcare delivery institutions) and NGO facilities were supposed to be offering national policy recommended anti-malarials for free, some were providing it at a cost. The reasons for this are not clearly understood, but it is believed that service providers are not adequately monitored by the appropriate authorities. Also it is possible that some NGOs might be operating on a cost recovery, non-profit basis, although this was not ascertained. To curb this practice, which undermines national antimalarial policy, performance based incentives for those in remote areas and the creation and use of logistics management information systems could be instituted.

Reassuringly, AS+AQ was mainly offered for free, however, where it was sold at a cost in the public sector, the price was comparable to that found in the private sector. Overall quinine for treatment of complicated malaria and SP for IPTp were comparably more affordable than first-line AS+AQ when they were sold. With out-of-pocket health care costs being 70% for Sierra Leoneans [18], innovative financing mechanisms should be considered within a strategy to increase equitable access in all sectors of the market. Studies have indicated that patients are more likely to seek treatment in the private rather than public sector given the travel distance, long waiting times, and poor availability of drugs in public sector facilities [16], [19]. Therefore if the proposed Global Fund’s Affordable Medicines Facility-malaria (AMFm) strategy [20] is successful in decreasing the price disparity by subsidizing high-quality anti-malarials all sectors of the market, there is a potential to increase widespread access to affordable ACTs.

There are several limitations to this study, similar to those encountered by the outlet survey study conducted in Burundi [13], The study lacked comparator drugs (e.g. other essential medicines), which made it difficult to understand whether other government fully subsidized essential medicines were also being sold at a price or if the findings were specific to the class of anti-malarials. In addition, it was unclear which specific anti-malarial was dispensed to patients when both policy and non-policy recommended anti-malarials were stocked in an outlet, especially when several private outlets stocked more than 7 brands of medicines. A “mystery shopper” survey should be conducted to evaluate any potential bias that may have occurred from direct questioning of medicine dispensers on their knowledge and practice. Furthermore, we cannot exclude the possibility of selection bias since it is unclear how the selection of medicine outlets within one day’s travel from the main urban public outlet in the region might under- or over-estimate the accessibility of policy recommended anti-malarials.

### Conclusions

Results of this study indicate that a relatively high proportion of public medicine outlets stock free first-line AS+AQ for treatment of uncomplicated malaria in Sierra Leone. With comparatively low proportions of private facilities stocking AS+AQ, expanding the involvement of the private sector towards the rational use of ACTs will be beneficial to the entire population.

The widespread availability of ACTs in the public sector can be expected to indirectly discourage the sale of monotherapies, counterfeit and substandard anti-malarial drugs by the private sector [21], [22], as well as provide confidence that the uptake of the newly introduced FDC ASAQ will be high in Sierra Leone. Effective measures are needed to strengthen the drug distribution system to control the widespread availability of banned chloroquine in the private sector in particular, and to counter the lack of availability and high cost of policy anti-malarials in the private sector, which continues to undermine the anti-malarial market throughout Africa [13], [23]–[25]. In addition to ensuring adequate supplies of ACTs, it is essential that the affected population is able to access health services, where available, for successful malaria control.
